# The Regulations of Insane Asylums

**Published:** 1874-03

**Authors:** 


THE REGULATION OF INSANE ASYLUMS.—A bill has been introduced into the New York Legislature on this subject, providing as follows:

"The Governor of the State is authorized to appoint for the City and County of New York not less than ten nor more than fifty, and in every other county not less than five nor more than twenty, reputable physicians, to be known as Examiners of Lunacy, and to fill vacancies occurring among such examiners as they occur; and in every case arising in any county requiring, as now provided by law, the certificate of two physicians to authorize the confinement for safe keeping of any person on the ground of lunacy, such certificate shall be required to be signed by two of the Examiners of Lunacy so appointed, resident in the county in which such case of lunacy is found; and no person shall be received as a lunatic in any asylum, public or private, without such certificate."

Up to the time of our going to press its fate is undecided. If the said bill should pass, and the proper persons be appointed on the commission, it may serve a good purpose. It is unnecessary to assume that such gentlemen should be well-educated alienists.

This being the case, we shall then have the initiative taken towards establishing a corps of experts on insanity, whose duty it may be to examine all cases of doubtful insanity that may come before the courts.—*Medical Record*

